# Supplementary material for: Mental Health and Physical Complaints of German Children and Adolescents before and during the COVID-19 Pandemic: A Repeated Cross-Sectional Study
Source: Int J Environ Res Public Health. 2023 Mar 2;20(5):4478. doi: 10.3390/ijerph20054478 (PMC10001698; doi:10.3390/ijerph20054478)
Supplement: Supplementary file 1 [file ijerph-20-04478-s001.zip › ijerph-2243454-Figure S1.pdf]

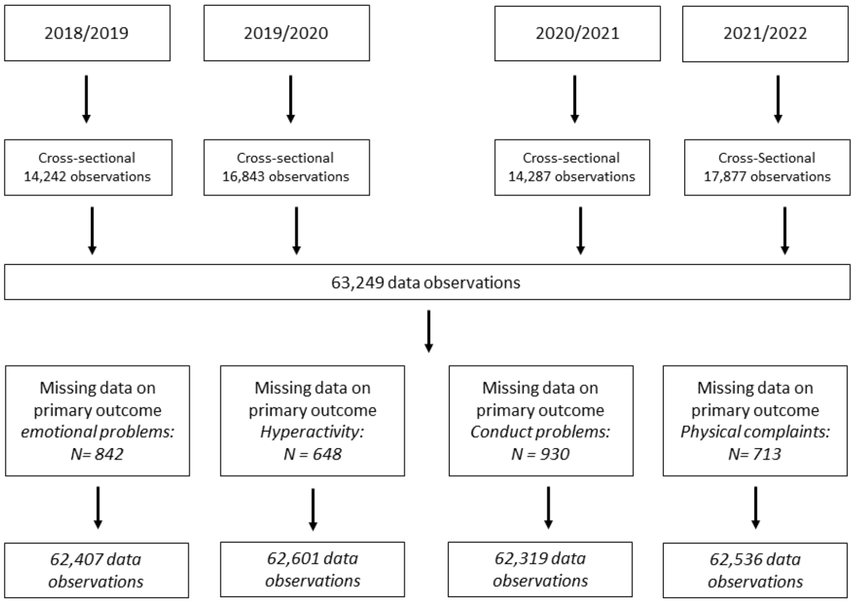

Figure S1. Selection into cross-sectional samples. Repeated cross- sectional analysis sample for each primary outcome, and missing data.
