# Supplementary material for: Mental Health and Physical Complaints of German Children and Adolescents before and during the COVID-19 Pandemic: A Repeated Cross-Sectional Study
Source: Int J Environ Res Public Health. 2023 Mar 2;20(5):4478. doi: 10.3390/ijerph20054478 (PMC10001698; doi:10.3390/ijerph20054478)
Supplement: Supplementary file 1 [file ijerph-20-04478-s001.zip › ijerph-2243454-Tables S1-S4.pdf]

Table S1. Mean emotional SDQ scores. Repeated cross-sectional analysis from 2018-2019 to 2021-2022 waves of "Präventionsradar", by year.

	2018-2019	2019-2020	2020-2021	2021-2022
N Total sample	13,964	16,681	14,191	17,571
	Mean emotional SDQ scores (95% CI)			
Total sample	2.89 (2.84-2.93)	2.96 (2.92-3.00)	3.29 (3.25-3.34)	3.48 (3.44-3.52)
Gender				
girl	3.69 (3.63-3.75)	3.85 (3.79-3.92)	4.23 (4.16-4.30)	4.46 (4.40-4.53)
boy	2.13 (2.08-2.19)	2.13 (2.08-2.18)	2.39 (2.33-2.44)	2.46 (2.41-2.52)
Age, years				
9-12	2.55 (2.49-2.61)	2.62 (2.56-2.68)	2.81 (2.74-2.87)	3.03 (2.97-3.10)
13-18	3.11 (3.06-3.17)	3.19 (3.13-3.24)	3.61 (3.55-3.68)	3.77 (3.71-3.83)

Table S2. Mean inattention-hyperactivity SDQ scores. Repeated cross-sectional analysis from 2018-2019 to 2021-2022 waves of "Präventionsradar", by year.

	2018-2019	2019-2020	2020-2021	2021-2022
N Total sample	14,056	16,754	14,201	17,590
	Mean inattention-hyperactivity SDQ scores (95% CI)			
Total sample	3.54 (3.51-3.58)	3.64 (3.61-3.68)	3.74 (3.70-3.77)	3.85 (3.82-3.89)
Gender				
girl	3.51 (3.46-3.56)	3.59 (3.54-3.63)	3.67 (3.62-3.73)	3.85 (3.81-3.90)
boy	3.59 (3.54 -3.64)	3.67 (3.62-3.72)	3.76 (3.70-3.81)	3.80 (3.75-3.85)
Age, years				
9-12	3.62 (3.56-3.67)	3.75 (3.69-3.80)	3.66 (3.60-3.72)	3.92 (3.86-3.97)
13-18	3.50 (3.45-3.54)	3.57 (3.53-3.62)	3.78 (3.73-3.83)	3.81 (3.76-3.85)

Table S3. Mean conduct problem SDQ scores. Repeated cross-sectional analysis from 2018-2019 to 2021-2022 waves of "Präventionsradar", by year.

	2018-2019	2019-2020	2020-2021	2021-2022
N Total sample	13,942	16,660	14,168	17,531
	Mean conduct problem SDQ scores (95% CI)			
Total sample	2.06 (2.03-2.09)	2.19 (2.16-2.22)	2.14 (2.11-2.17)	2.13 (2.11-2.16)
Gender				
girl	1.95 (1.91-1.99)	2.06 (2.02-2.10)	2.02 (1.98-2.07)	2.06 (2.02-2.10)
boy	2.17 (2.13-2.22)	2.27 (2.23-2.31)	2.21 (2.16- 2.25)	2.15 (2.11-2.19)
Age, years				
9-12	1.92 (1.87-1.96)	2.11 (2.07-2.15)	2.07 (2.03-2.12)	2.12 (2.08-2.17)
13-18	2.15 (2.11-2.19)	2.24 (2.20-2.28)	2.17 (2.13-2.21)	2.13 (2.09-2.16)

Table S4. Mean physical complaints scores. Repeated cross-sectional analysis from 2018-2019 to 2021-2022 waves of "Präventionsradar", by year.

	2018-2019	2019-2020	2020-2021	2021-2022
N Total sample	14,075	16,753	14,178	17,530
	Mean physical complaints scores (95% CI)			
Total sample	1.76 (1.75-1.78)	1.76 (1.75-1.78)	1.76 (1.74-1.77)	1.93 (1.91-1.94)
Gender				
girl	1.92 (1.89-1.94)	1.95 (1.93-1.98)	1.96 (1.94-1.99)	2.17 (2.15-2.20)
boy	1.61 (1.59-1.64)	1.57 (1.55-1-59)	1.55 (1.53-1.57)	1.66 (1.64-1.68)
Age, years				
9-12	1.64 (1.62-1.66)	1.63 (1.61-1.65)	1.58 (1.55-1.60)	1.74 (1.72-1.77)
13-18	1.84 (1.81-1.86)	1.86 (1.84-1.88)	1.87 (1.85-1.89)	2.04 (2.02-2.06)
